# Vorinostat and hydroxychloroquine improve immunity and inhibit autophagy in metastatic colorectal cancer

**DOI:** 10.18632/oncotarget.10824

**Published:** 2016-07-23

**Authors:** Sukeshi Patel, Vincent Hurez, Steffan T. Nawrocki, Martin Goros, Joel Michalek, John Sarantopoulos, Tyler Curiel, Devalingam Mahalingam

**Affiliations:** ^1^ Cancer Therapy and Research Center, University of Texas Health Science Center at San Antonio, San Antonio, TX, USA

**Keywords:** vorinostat, hydroxychloroquine, colorectal cancer, autophagy, immunity

## Abstract

Hydroxychloroquine (HCQ) enhances the anti-cancer activity of the histone deacetylase inhibitor, vorinostat (VOR), in pre-clinical models and early phase clinical studies of metastatic colorectal cancer (mCRC). Mechanisms could include autophagy inhibition, accumulation of ubiquitinated proteins, and subsequent tumor cell apoptosis. There is growing evidence that autophagy inhibition could lead to improved anti-cancer immunity. To date, effects of autophagy on immunity have not been reported in cancer patients. To address this, we expanded an ongoing clinical study to include patients with advanced, refractory mCRC to evaluate further the clinical efficacy and immune effects of VOR plus HCQ. Refractory mCRC patients received VOR 400 milligrams orally with HCQ 600 milligrams orally daily, in a 3-week cycle. The primary endpoint was median progression-free survival (mPFS). Secondary endpoints include median overall survival (mOS), adverse events (AE), pharmacodynamic of inhibition of autophagy in primary tumors, immune cell analyses, and cytokine levels. Twenty patients were enrolled (19 evaluable for survival) with a mPFS of 2.8 months and mOS of 6.7 months. Treatment-related grade 3–4 AEs occurred in 8 patients (40%), with fatigue, nausea/vomiting, and anemia being the most common. Treatment significantly reduced CD4^+^CD25^hi^Foxp3^+^ regulatory and PD-1^+^ (exhausted) CD4^+^ and CD8^+^ T cells and decreased CD45RO-CD62L^+^ (naive) T cells, consistent with improved anti-tumor immunity. On-study tumor biopsies showed increases in lysosomal protease cathepsin D and p62 accumulation, consistent with autophagy inhibition. Taken together, VOR plus HCQ is active, safe and well tolerated in refractory CRC patients, resulting in potentially improved anti-tumor immunity and inhibition of autophagy.

## INTRODUCTION

Chemotherapy and novel biologics targeting the epidermal growth factor receptor (EGFR) and vascular endothelial growth factor (VEGF) pathways have been the mainstay to treat advanced or metastatic colorectal cancer (mCRC). However, in heavily treated, advanced refractory CRC patients, these agents only modestly improve survival [1]. Currently, the median survival of patients with mCRC following failure of 5-florouracil (5FU)-based chemotherapy and anti-VEGF and/or anti-EGFR therapy is approximately 6 months [2]. It is postulated that primary or acquired resistance to these therapeutics likely accounts for dismal outcomes.

Tumor autophagy induction has been shown following treatment with chemotherapeutic agents and/or novel biologics and could significantly contribute to resistance to a number of anticancer therapeutic modalities [3, 4]. The activation of stress response genes, such as p53, by anticancer therapies can stimulate autophagy in addition to apoptosis [5]. Although prolonged autophagy can result in cancer cell death, recent investigations suggest that therapy-induced autophagy promotes cancer cell survival, and thus, could diminish the efficacy of some agents [6–8]. Further, there has been growing evidence that induction of autophagy could result in immune evasion of tumors that can reduce therapeutic efficacy [9]. Currently, a clear understanding of the role of autophagy and its effects on tumor immunity in the clinic is lacking. A better understanding could help develop more effective therapeutic options.

Autophagy is a “double-edged sword” as it has a dual role in the initiation of tumor suppression and promotion of tumor survival, as well as autophagy activates immune response yet impairs anti-immunity at other times [10, 11]. Autophagy is needed for cell survival and influences innate and adaptive immunity through its effects on antigen presentation, naive T cell repertoire selection, and T cell homeostasis, among many other immune effects [12]. Increased autophagy in tumor cells prevents effector cell mediated cytotoxicity [13]. Stress-induced release of the damage-associated molecular pattern molecule high-mobility group box 1 (HMGB1) induces cytoprotective autophagy and once extruded into the extracellular matrix recruits regulatory T cells [13] that reduce anti-tumor immunity [14]. Within antigen presenting cells (APCs), antigen processing and delivery to the major histocompatibility complex (MHC) is directed by the proteasome and autophagy. Tumor cells can expel autophagic contents into the extracellular matrix from tumor cells, providing antigens to dendritic cells for T cell priming [13]. Therefore, for improved anti-tumor activity, crippling the protective effects of autophagy in cancer cells and suppressive immune cells without inhibiting the essential functions of autophagy in the defensive antitumor immune cells is an attractive strategy. However, there is essentially nothing reported on autophagy effects on anti-cancer immunity in humans.

Histone deacetylase (HDAC) inhibitor-induced autophagy blunts its anticancer activity [8]. The combination of the HDAC inhibitor, vorinostat (VOR) and the autophagy inhibitor, chloroquine (CQ) each increase lysosomal protease cathepsin D (CTSD), a key mediator of pro-apoptotic cell death [3, 4]. We showed that VOR plus hydroxychloroquine (HCQ) significantly increased intra-tumoral p21, cathepsin D, and LC3B in a phase I trial, consistent with autophagy inhibition [15]. Data is conflicting regarding VOR effects on immunity. *In vitro* studies have shown anti-inflammatory properties of HDAC inhibitors on human peripheral blood mononuclear cells (PBMCs) via suppression of cytokines, such as TNF-α and IL-1β [16]. *In vitro* studies also demonstrated that HDAC inhibitors, including VOR, increase activating natural killer (NK) receptors expressing on tumor cells, promoting PBMCs induced tumor cell death [17]. In other studies, VOR alone depresses NK cell activity and inhibits APC activation and interferon-α (IFN-α) production by plasmacytoid dendritic cells [18, 19]. In the peripheral blood samples of Hodgkin lymphoma patients, suppression of T cell programmed death 1 (PD-1) expression after treatment with the pan-HDAC inhibitor panobinostat was observed [20]. However, data are lacking regarding the effect of autophagy on immunoregulation in the clinical setting.

In our phase 1 dose escalation trial, 600 milligrams (mg) of HCQ and 400 mg of VOR by mouth (PO) daily was established as the maximum tolerated dose (MTD) and recommended phase II regimen (RPD2) in mCRC patients [15]. However, immunity was not evaluated. Therefore, to evaluate immune effects following autophagy modulation, as well as the clinical efficacy and safety of the combination of VOR and HCQ in patients with mCRC, we designed a single-arm expansion cohort of HCQ plus VOR in patients with refractory mCRC. Our hypothesis was that VOR plus HCQ would improve clinical efficacy and anti-tumor immunity.

## RESULTS

Patients with refractory mCRC (failing all standard therapies) were enrolled onto a single-arm expansion cohort to assess the efficacy, safety and effects on immunity of VOR 400 mg PO and HCQ 600 mg PO daily, in a 3-week cycle.

### Patient characteristics

Twenty patients were enrolled at the Cancer Therapy and Research Center, San Antonio, Texas, from December 2012 to July 2014 (Table 1). The mean age was 61 years (range 44–74). Thirty-five percent were female and 65% were male. Forty-five percent were Caucasian, and 50% were Hispanic. Ninety percent of patients were ECOG 0–1. Ninety percent were colon primary, whereas 10% were rectal primary; 55% were KRAS mutated. Sixty-five percent had received three or more prior treatment lines, of which 20% had received regorafenib (Table 2). Thirty-five percent required dose reduction of either drug on study.

**Table 1:** Demographics of patients with refractory mCRC receiving VOR plus HCQ

		*N* = 20
**Mean age**		61 (44–74)
**Sex**	Female	7
	Male	13
**Ethnicity**	Caucasian	9
	Hispanic	10
	Black	1
**ECOG**	0	5
	1	13
	2	2
**Site**	Colon	18
	Rectum	2
**KRAS**	Wild type	9
	Mutated	11
**Prior lines of treatment**	0–1	0
	2	7
	3+	13
	Previous Regorafenib	4
**Dose Reduction on Study**	Yes	7
	No	13

*N* = number.

**Table 2:** Patient characteristics

Patient Number	Age	Sex	Ethnicity	Site	KRAS	Organ Metastases	Lines of prior Chemo	Prior regorafenib	ECOG	Cycles	Dose Reduction
33	64	Female	Hispanic	rectum	WT	5	4	no	1	10	yes
34	69	Female	Hispanic	colon	mutated	2	2	no	2	*	no
35	65	Female	White	colon	WT	3	5	yes	1	2	no
36	45	Male	White	colon	WT	1	3	no	1	8	no
37	72	Male	Hispanic	colon	mutated	1	2	no	1	2	no
39	45	Female	Black	colon	mutated	1	3	yes	2	2	yes
41	74	Male	White	colon	mutated	1	2	no	0	2	no
42	70	Female	Hispanic	colon	mutated	4	2	no	1	3	yes
44	64	Female	Hispanic	colon	WT	2	4	no	1	2	yes
45	61	Male	Hispanic	colon	WT	3	4	no	1	1	no
46	58	Male	White	colon	WT	2	7	yes	1	10	no
51	53	Female	White	colon	mutated	1	3	no	0	2.3	yes
52	60	Male	White	colon	mutated	2	3	no	0	4	no
54	44	Male	White	colon	WT	2	3	no	1	7	yes
55	58	Male	Hispanic	colon	mutated	3	3	yes	1	1	no
56	59	Male	White	colon	WT	2	3	no	1	2	no
58	51	Male	Hispanic	colon	mutated	1	3	no	0	2	no
59	59	Male	Hispanic	rectum	mutated	2	2	no	1	2	yes
60	67	Male	Hispanic	colon	WT	3	2	no	1	2	no
61	61	Male	White	colon	mutated	2	2	no	0	12	no

* Patient 34 not evaluable for survival because the patient received only 1 day of treatment and went to hospice on Cycle 1, Day 8. WT = wild type.

### Efficacy

**Figure 1:**
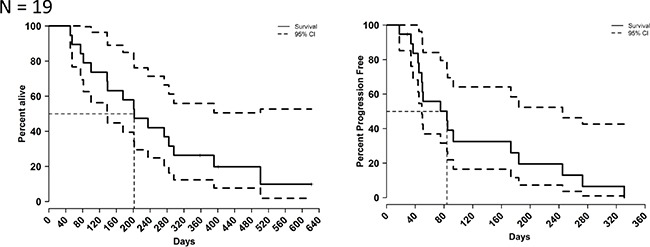
Efficacy of VOR plus HCQ Five patients (26%) had stable disease ≥ 16 weeks. mPFS = median progression-free survival. mOS = median overall survival.

**Figure 2:**
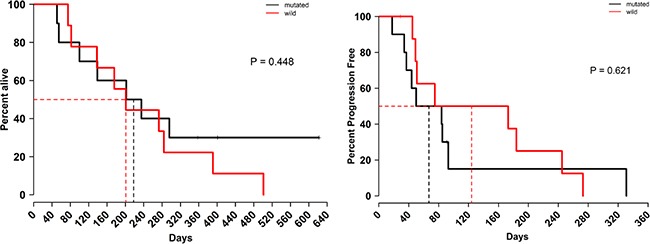
Efficacy of VOR plus HCQ according to KRAS status (Left) median overall survival. (Right) median progression-free survival.

Median cycle number was 2, and 19 patients were evaluable for response (one patient received 1 day of treatment and went off study on cycle 1 day 8 as he went to hospice). Seventeen patients went off study due to disease progression (radiographical and/or clinical), and two patients went off study due to toxicity. Five patients had stable disease for more than 18 weeks. No partial or complete responses were observed. The median progression-free survival (PFS) was 2.8 months (95% CI: 1.63–8.16). The median overall survival (OS) was 6.7 months (95% CI: 4.63-NR) (Figure 1). No differences in survival were seen based on KRAS status (Figure 2).

### Toxicity

Nineteen (95%) patients had treatment-related toxicities, mostly grade 1–2. Treatment was well-tolerated with manageable non-hematological toxicities including fatigue (*n* = 11, 55%) and nausea/vomiting (*n* = 13, 65%). The most common hematologic toxicities included anemia (*n* = 15, 75%) and thrombocytopenia (*n* = 8, 40%). Treatment-related grade 3 adverse events (AEs) were nausea/vomiting (*n* = 3) and anemia (*n* = 3). Three (15%) patients had grade 4 thrombocytopenia, and grade 4 international normalized ratio (INR) elevation occurred in one patient on warfarin. No grade 5 AEs were observed. (Table 3).

**Table 3:** Adverse events in patients receiving VOR plus HCQ

Adverse Event	0	1	2	3	4^*^
Fatigue	9	8	3	-	-
Nausea/vomiting	7	5	5	3	-
Stomatitis	19	1	-	-	-
Anorexia	17	2	1	-	-
Diarrhea	18	2	-	-	-
Weight Loss	19	-	1	-	-
Blurred Vision	19	1	-	-	-
Leukopenia	16	3	1	-	-
Neutropenia	19	1	-	-	-
Anemia	5	9	3	3	-
Thrombocytopenia	12	5	-	-	3

Adverse events graded by CTCAE version 3.0 guidelines.

* Grade 4 international normalized ratio(INR) elevation occurred in one patient on Coumadin.

### Pharmacodynamic analysis

**Figure 3:**
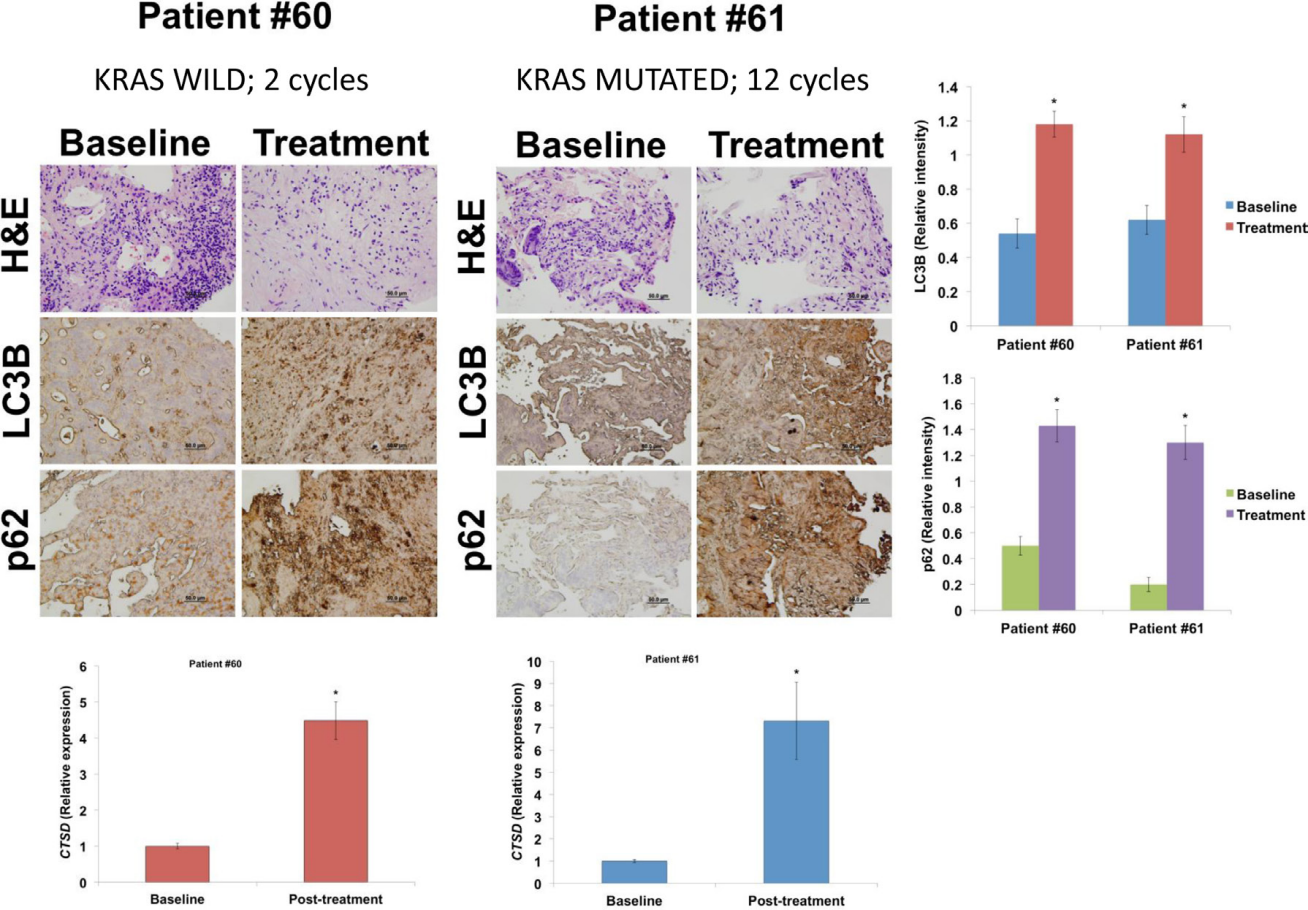
HCQ and VOR increase the expression levels of LC3B, p62, and cathepsin D (Top) Tumor biopsies were collected at baseline and following one cycle of treatment. LC3B and p62 levels were measured by immunohistochemistry. Relative intensity of staining was determined by densitometry. Mean ± SD, *n* = 3. *Indicates a significant difference from baseline, *p* < 0.05. (Bottom) qRT-PCR for cathepsin D (*CTSD*) was performed on snap-frozen tumor biopsies. *CTSD* expression was significantly increased in post-treatment specimens. Mean ± SD, *n* = 2, *p < 0.05.

As tumor biopsies were optional in this expansion phase, two patients agreed to on-study biopsies. Two on-study biopsies (patient #60 and #61) were performed after cycle 1. Compared to baseline biopsies, we observed increased LC3B and p62 protein by immunohistochemistry, consistent with treatment with HCQ (Figure 3, top). p62 accumulation is a key marker of autophagy inhibition. We also conducted qRT-PCR for *CTSD* and noted a significant increase following treatment in both patients (Figure 3, bottom). We had previously identified this gene as an important marker of activity following treatment with HCQ plus VOR in our preclinical models [3, 4].

### Immune analysis

**Figure 4:**
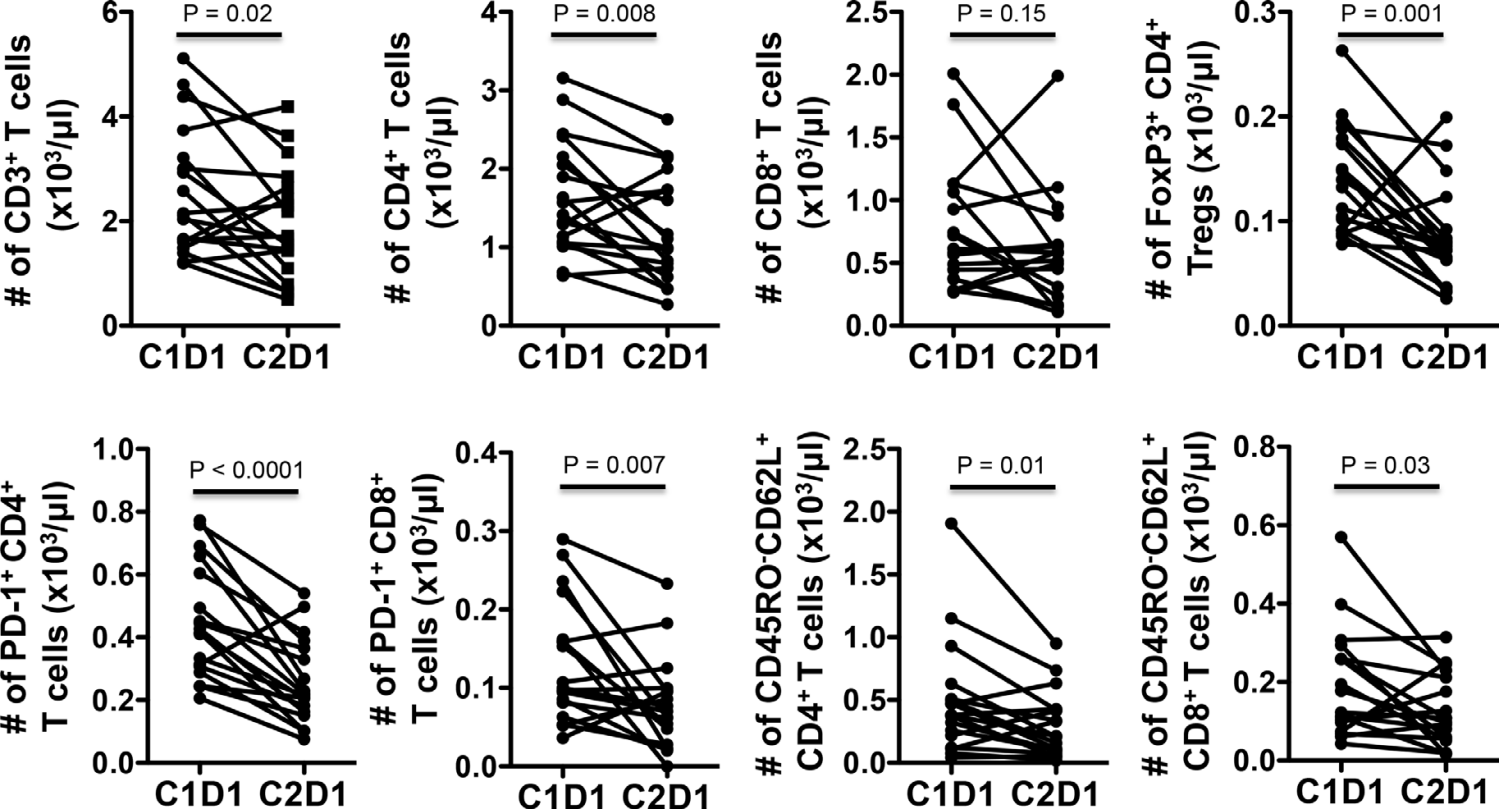
VOR plus HCQ treatment in mCRC results in reduction in T cell, regulatory T cells, exhaustion markers, and in naïve CD4^+^ T cell phenotype Flow cytometry analyses of absolute numbers of various T cell populations (CD3+, CD4+, CD8+, regulatory T cells) and surface markers (PD-1, CD45RO, CD62L) in total PBMCs for each individual patient at baseline and after cycle one. *P* values, paired *t*– test.

**Figure 5:**
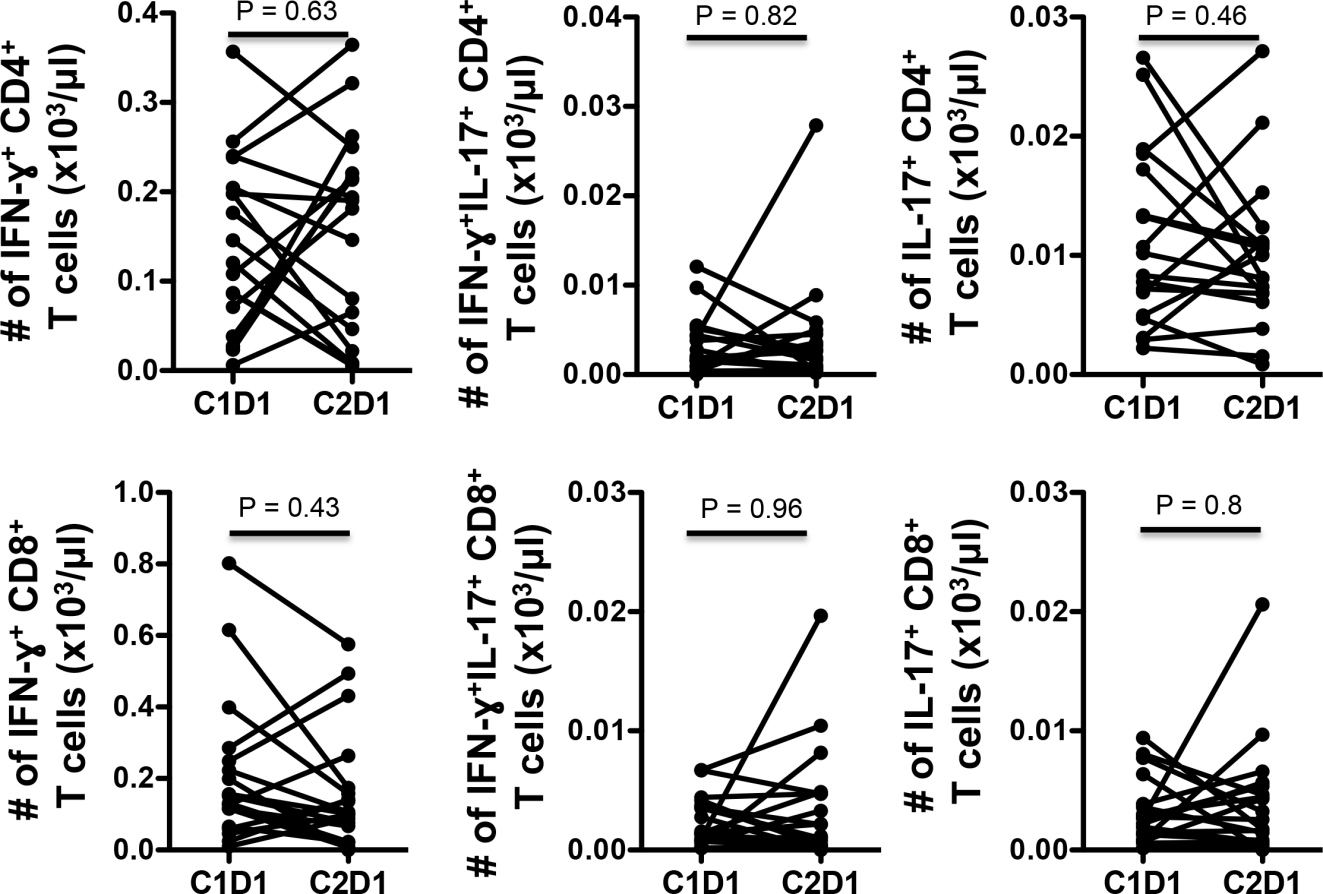
VOR plus HCQ treatment in mCRC did not result in observed significant changes in intracellular cytokines across patients Flow cytometry analyses of absolute numbers of IFN-γ^+^ and IL-17^+^ T cell for each individual patient at baseline and after cycle one. Intracellular cytokine staining after 5 hour PMA/ionomycin stimulation. *P* values, paired *t*– test.

**Figure 6:**
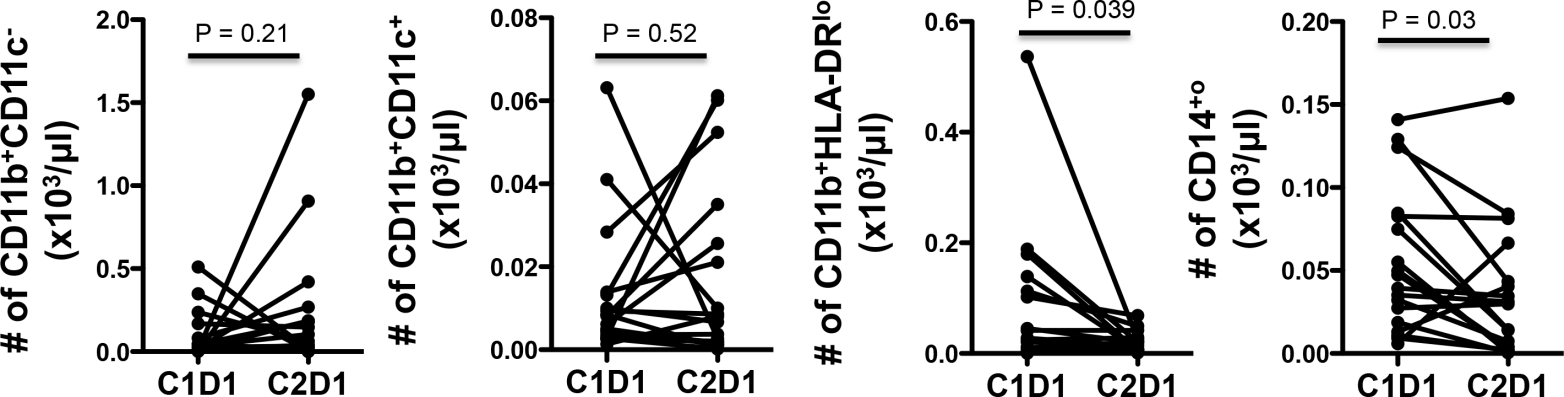
VOR plus HCQ treatment in mCRC decreased MDSCs (CD11b^+^HLA-Dr^lo^) and monocytes (CD14^+^) Flow cytometry analyses of absolute numbers of various myeloid cell populations in total PBMCs for each individual patient at baseline and after cycle one. *P* values, paired *t*– test.

Flow cytometry (FACS) of peripheral blood mononuclear cells (PBMCs) were done at baseline and after one cycle of treatment. Treatment significantly reduced peripheral blood T cells (CD3^+^) but did not change the proportion of CD4 or CD8 among those T cells (Figure 4, Supplementary Figure 1). There was a significant reduction in the proportion of regulatory T cells (CD25^+^FoxP3^+^) among CD4^+^ T cells (Figure 4), which are considered deleterious in cancer by suppressing active anti-tumor immunity. PD-1, an inhibitory co-signaling molecule regarded as a marker for exhausted, poorly functional CD4^+^ and CD8^+^ T cells, often highly expressed in tumor infiltrating T lymphocytes, was also reduced in the peripheral CD4^+^and CD8^+^ T cells, suggesting improved immune response (Figure 4). We also saw a slight decrease in CD45RO^−^CD62L^+^ CD4^+^ and CD8^+^T cells, populations usually defined as naive T cells, again consistent with increased T cell activation (Figure 4). We assessed cytokine producing cells using intracellular flow cytometry on stimulated PBMCs but could not find significant changes in IFN-γ or IL17 expressing CD4^+^ or CD8^+^ T cells across patients (Figure 5). We also assessed various peripheral myeloid population and saw a decrease in myeloid derived suppressor cells (defined as CD11b^+^HLA-DR^lo^) and monocytes (CD14^+^) but no significant changes in other CD11b^+^ myeloid cells, dendritic cells (CD11b^+^CD11c^+^) or in CD19^+^ B cells (Figures 6 and data not shown).

## DISCUSSION

We found that the combination of VOR plus HCQ is active, safe and well tolerated in refractory CRC patients. The most common toxicities were fatigue, nausea/vomiting, anemia and thrombocytopenia. VOR plus HCQ elicited a median PFS of 2.8 months and median OS of 6.7 months, with five patients having stable disease for more than 16 weeks. Survival of this combination is comparable to other oral drugs used for refractory colorectal cancer, including regorafenib (median OS of 6.4 months; median PFS of 1.9 months) and TAS-102 (median OS of 7.1 months, median PFS of 2 months) [2, 22]. No patients had a complete or a partial response in this early phase study; whereas, larger randomized studies of TAS-102 and regorafenib showed response rates of 1.6% and 1%, respectively [2, 22]. Given the favorable toxicity profile and efficacy of this novel regimen, VOR plus HCQ could be an alternative treatment for refractory colorectal cancer. This study is limited due to the small sample size and lack of a comparator arm; therefore, a randomized phase II study will evaluate the efficacy of VOR plus HCQ in comparison to regorafenib.

VOR plus HCQ's known effects on autophagy were observed in this cohort. On-study biopsies showed increased expression of the lysosomal protease cathepsin D and accumulation of LC3B and p62, consistent with autophagy inhibition. These findings were associated with reduced tumor autophagy by VOR plus HCQ. Additionally, these effects were observed in a patient with wild-type K-RAS as well as a patient with mutant K-RAS, indicating activity in both tumor types. Furthermore, markers of autophagy inhibition were increased in paired biopsies obtained from two patients enrolled in this expansion study, one of whom had prolonged disease stabilization for 12 cycles. The other, however, progressed following 2 cycles. These findings are consistent with observations from the phase I study [15], and further correlation of these biomarkers with efficacy should be evaluated in larger studies, along with other potential biomarkers of efficacy. For example, HMGB1 increases with autophagy in CRC, and HMGB1 acts upon binding to RAGE (receptor for advanced glucated end products) [23, 24], which could be a marker of autophagy.

Given the lack of the comparator arm in a phase Ib study, we cannot precisely determine if the immune effects are due to VOR alone or the addition of HCQ enhances the effect of VOR. Few clinical trials with HDAC inhibitors and the effect on immunity have resulted; however, one study with belinostat plus three chemotherapeutic agents for thymic tumors demonstrated declines in regulatory T cells and exhausted T cells [25]. Further evaluation of CDKN1a, a marker of VOR, in subsets of T cells are indicated in future studies.

Autophagy has been implicated in both cell survival and cell death in T cells. In addition to effects of VOR plus HCQ on autophagy, alterations in immune responses were observed. Treatment with VOR plus HCQ resulted in decreased phenotypic regulatory T cells, markers of T cell exhaustion (PD-1), and naive T cells, yet increased effector memory T cells. This pattern is consistent with improved anti-tumor immunity [26]. These immune effects will be corroborated with functional confirmatory immune studies in follow up. Autophagy's role is dependent on the cell type, and therefore, autophagy inhibition can alter the balance of immune subsets [27]. For example, Beclin 1-deficient Th1 cells are more susceptible to cell death, with accumulation of procaspase-8/p62 protein complex in Beclin 1-deficient T cells, suggesting a role of autophagy in T cells [27]. Also, autophagy is active in regulatory T cells, leading to survival and resistance; whereas, autophagy deficiency results in defective regulatory T cell function [28]. This merits additional investigations with VOR plus HCQ in our ongoing study. In this study, the immune effects did not correlate with clinical disease stability; therefore, future studies should correlate the immune changes in the peripheral blood in comparison to the immune effects in the patient's tumor.

Predictive markers for response to immunotherapy, such as expression of apoptosis-associated molecules such as FAS, could better identify patients with optimal benefit from VOR plus HCQ [29]. To evade Fas-mediated apoptosis, cancer cells can down-regulate Fas, which is a hallmark of metastatic human colorectal cancer [30]. VOR-based therapy up-regulates Fas expression in the metastatic human colon carcinoma cells [31]. VOR plus HCQ could preferentially decrease regulatory T cells over effector memory T cells through Fas effects. Regulatory T cells are highly sensitive to Fas-mediated apoptosis, whereas effector T cells are relatively resistant [32]. Thus, Fas-based cancer therapy might not only induce tumor cell apoptosis but also induce regulatory T cell apoptosis to eliminate regulatory T cell-mediated immune suppression. Future studies with VOR plus HCQ in CRC should investigate the role of Fas- mediated immune cell apoptosis in relation to clinical effects.

Markers of T cell exhaustion, including PD-1, were decreased with VOR plus HCQ in CRC patients. Tumor PD-1 ligand (PD-L1) plays an important role in tumor immune evasion. It is immunosuppressive through engaging T cell PD-1 [33], and thus our combination could reduce immunosuppressive effects through reducing T cell PD-1. Mismatch repair-deficient CRC (about 15% of all CRC) [34, 35] is highly immunogenic and responsive to immune checkpoint blockade versus mismatch repair-proficient CRC [36]. The increased number of mutation-associated neoantigens in mismatch repair-deficient CRC leads to enhanced anti-PD-1 responsiveness, which is not observed in mismatch repair-proficient CRC [37]. In our study, tumor mismatch repair status was not available for many patients as this was not standard of care at the time of enrollment. As a result, correlative studies with mismatch repair status and efficacy of VOR plus HCQ were not feasible in all patients; however, the two patients who received on-study tumor biopsies did have mismatch repair-proficient tumors. Our ongoing study with VOR plus HCQ will evaluate efficacy based on tumor mismatch-repair status and PD-L1 expression and other emerging biomarkers of immune-responsive cancers. This would guide further investigation of VOR plus HCQ in a subset of patients or in combination with immune modulators, such as anti-PD1 inhibitors, to enhance efficacy

This study further supports the need to understand better the complex interplay between autophagy modulation and immunity in cancers, including CRC. A better understanding of these integrated systems is imperative for future drug development of anti-cancer therapies. A randomized phase II trial of VOR/HCQ versus regorafenib is now open to enrollment.

## MATERIALS AND METHODS

### Patient population

Patients at least 16 years of age, with histologically or cytologically confirmed colon adenocarcinoma who progressed despite standard therapy or for whom no standard therapy was available were eligible. Patients must have been treated in the past with irinotecan and/or oxaliplatin and/or anti-VEGF/EGFR therapy or intolerant to these agents. KRAS mutational status was documented and no prior treatment with VOR or HCQ was permitted. Other key inclusion criteria included measurable or evaluable disease defined by RECIST 1.0 [38]. All patients met the following inclusion criteria: Eastern Cooperative Oncology Group performance status ≤ 2; adequate bone marrow, liver, and kidney function (*i.e.,* absolute neutrophil count ≥ 1000/mm^3^, platelets ≥ 75,000/mm^3^); creatinine ≤ 2 times the upper limits of normal; total bilirubin ≤ 1.5 mg/dL; alanine aminotransferase and aspartate aminotransferase ≤ 3 times above the upper limits of the institutional normal alanine aminotransferase (aspartate aminotransferase can be < 5 times upper limits of normal if patients have hepatic involvement). Patients were excluded if they had one or more of the following conditions: previously documented macular degeneration or diabetic retinopathy, uncontrolled brain metastases, baseline QTc > 500 ms, clinically significant symptomatic hypercalcemia, or gastrointestinal dysfunction that might impair oral absorption. Patients with active, clinically significant and/or uncontrolled medical conditions were also excluded, including uncontrolled psoriasis.

### Protection of human research subjects

All patients provided written informed consent before enrollment. This study followed the ethical principles of the Declaration of Helsinki, the International Conference on Harmonization Guidelines for Good Clinical Practice, and local regulations (European Directive 2001/20/EC and US Code of Federal Regulations Title 21). The original protocol and all subsequent amendments were approved by the Institutional Review Board at the University of Texas Health Science Center at San Antonio.

### Study design

We designed a single-arm expansion cohort of HCQ plus VOR in patients with refractory mCRC. The RP2D of VOR 400 mg PO daily and HCQ 600 mg PO daily was determined by the phase I study of VOR and HCQ in solid tumors [21], and this dose was used for the single-arm expansion cohort. Three weeks of treatment (21 days) was defined as one cycle of therapy. Cycles were repeated without interruption, if drug tolerance was acceptable. If toxicity occurred, treatment holidays were allowed at the discretion of the principal investigator. Patients were allowed to continue on study as long as they had a clinical benefit (response or stable disease) and good tolerability profile. Patients went off treatment for disease progression, unacceptable toxicity, patient's refusal to continue treatment, or if the physician did not believe treatment was in the patient's best interest. All patients were followed every two months for survival.

### Study endpoints

Our major objectives were to determine efficacy of this combination and further evaluate the safety. We also assessed the pharmacodynamics of HCQ and VOR in peripheral blood mononuclear cells and effects on immunity. The primary endpoint was median PFS. Secondary endpoints were median OS, AEs, flow cytometry of PBMCs. In this expansion cohort, tumor biopsies were optional. Patients were evaluable for survival, if they received at least 1 cycle, or 3 weeks, of treatment.

### Efficacy and safety evaluations

Safety (AEs) was assessed according to CTCAE version 3.0 guidelines [39]. Assessments included regular laboratory evaluations, physical examinations, ECOG performance status, vital signs, weight, and periodic electrocardiogram recordings. All patients were monitored for safety from the first dose until 28 days following the final dose. Additional monitoring included baseline ophthalmologic evaluation that was repeated if any visual disturbances occurred while a patient was on study. All potential sites of tumor lesions were evaluated by CT and/or magnetic resonance imaging at baseline and every 6 weeks (2 cycles). Antitumor activity was determined according to RECIST 1.0 [38].

### Quantitative RT-PCR analyses

Total RNA was isolated from tumor cells that were snap-frozen following biopsy using the RNeasy Plus Mini Kit (Qiagen Inc., 74104). RNA was treated with the TURBO DNA-free™ Kit (Ambion Inc., AM1907). First-strand cDNA synthesis was performed with the high capacity cDNA Reverse Transcription Kit (Applied Biosystems, 4368813). *CTSD* transcripts were amplified using TaqMan^®^ Gene expression assays as previously described [15]. The relative expression of each gene was calculated with the 2^−ΔCt^ method using *GAPDH* as a housekeeping gene [15].

### Immunohistochemistry

Pre- and post-treatment tumor biopsies were collected from 2 patients enrolled on this study. Tumor biopsies were fixed in formalin and subsequently embedded in paraffin. Paraffin-embedded tumor sections were deparaffinized in xylene, exposed to a graded series of alcohol, and rehydrated in PBS (pH 7.5). Heat-induced epitope retrieval was performed by microwaving slides in a citrate buffer for 5 min. A 3% hydrogen peroxide solution in methanol was used to block endogenous peroxides. Slides were then incubated in a protein block solution (5% horse serum and 1% goat serum (Gibco, 16050 and 16210) in PBS (Corning Cellgro, 21–031-CV) for 20 min. Slides were exposed to LC3B and p62 antibodies diluted in the protein block solution at 4°C overnight as previously described [15]. After washing with PBS, slides were incubated with anti-rabbit secondary antibody conjugated to HRP (Jackson Immunoresearch, 111–035–003) for 1 hour at ambient temperature. Slides were exposed to 3,3′-diaminobenzidine (Dako, S1967) for 15 min to visualize positive reactions. Slides were rinsed with water and then briefly counterstained with Gill's hematoxylin solution (Sigma, GHS1128). Images were captured under 20× magnification with an Olympus fluorescent microscope equipped with a DP71 camera (Olympus, Center Valley, PA). MediaCybernetics Image-Pro Plus software Version 6.2.1 was used for image acquisition. ImageJ software was used for quantification of LC3B and p62 expression by densitometric analysis of three random fields containing viable tumor cells.

### Immune analysis

Whole blood samples from peripheral venipuncture or indwelling port were collected in sterile tubes containing lithium heparin, on cycle 1 day 1 (C1D1, baseline) and again on cycle 2 day 1 (C2D1). Total PBMCs were isolated using Ficoll-Paque Plus (GE Healthcare, Piscataway, NJ). 3 × 10^6^ PBMCs were labeled in PBS-1% FCS-EDTA using anti-CD3, -CD4, -CD11c, -CD25, (BD Biosciences, San Jose, CA), -CD8 (Invitrogen, Carlsbad, CA), -CD11b, -CD14, -CD45RO, -CD45RA, -CD62L (BioLegend, San Diego, CA), -CD127, -HLA-DR, -FoxP3, -PD-1, -IL-17A, or -IFN-γ (eBioscience, San Diego, CA) monoclonal antibodies, For cytokine detection, 3 × 10^6^ PBMCs were stimulated for 5 hours with Leukocyte Activation kit (BD bioscience), stained for surface markers, permeabilized using BD Fix/Perm protocol and stained intracellular cytokines. Cells were acquired using a BD LSRII flow cytometer (BD Biosciences), flow data analyzed using FlowJo (Ashland, OR) and statistics done with GraphPad Prism software (La Jolla, CA).

### Statistical analysis

Survival distributions were summarized with Kaplan-Meier curves and confidence bands. The significance of subgroup contrasts with regard to survival were assessed with log rank tests and changes in percent T cells, regulatory T cells, naïve CD4^+^ T cell phenotype, MDSCs (CD11b^+^HLA-Dr^lo^) and monocytes were assessed with paired *t*-tests. All statistical testing was two-sided with a significance level of 5%. Based on an exponential failure model with 52 weeks of follow-up and one sample 2-sided testing with a significance level of 5% with a historical median progression-free survival of 7 weeks, this study achieved 80% power with *N* = 16 subjects if the median progression-free survival after treatment was 14 weeks. To allow for a 10% loss to follow-up, the total number of patients required was 18.

## SUPPLEMENTARY MATERIALS FIGURE


