# Supplementary material for: Vorinostat and hydroxychloroquine improve immunity and inhibit autophagy in metastatic colorectal cancer
Source: Oncotarget. 2016 Jul 23;7(37):59087–97. doi: 10.18632/oncotarget.10824 (PMC5312297; doi:10.18632/oncotarget.10824)
Supplement: Supplementary file 1 [file oncotarget-07-59087-s001.pdf]

Vorinostat and hydroxychloroquine improve immunity and inhibit autophagy in metastatic colorectal cancer

Supplementary Materials

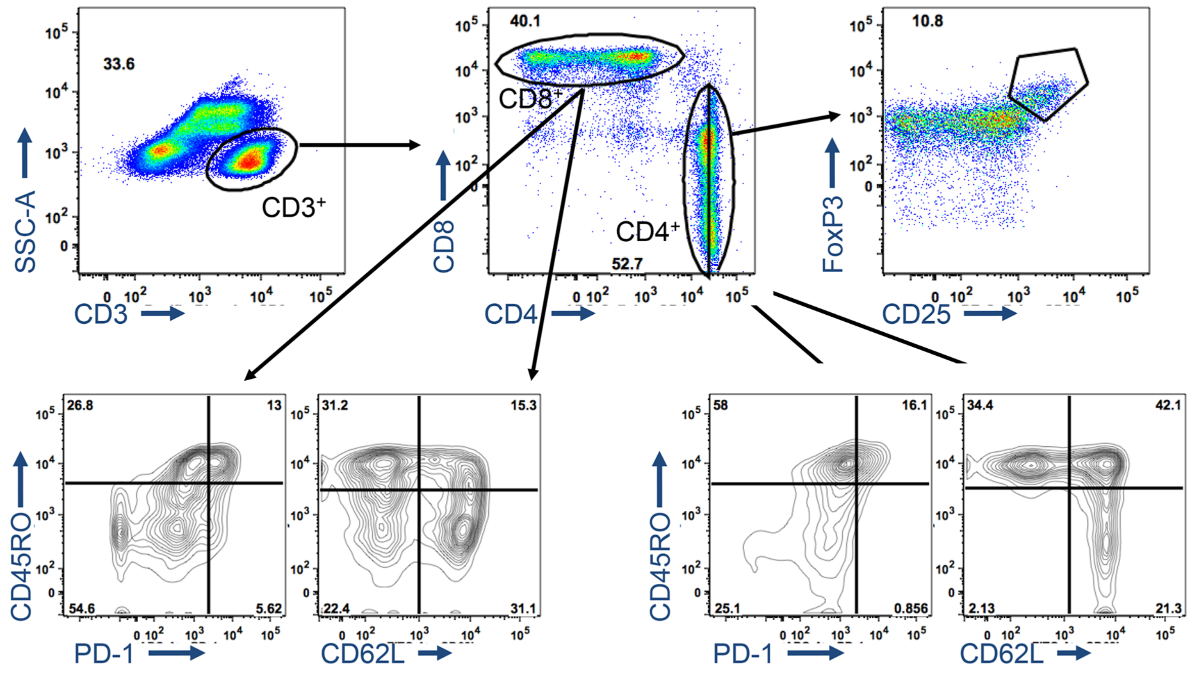

Supplementary Figure S1: Gating strategy for T cell analyzes. Representative flow cytometry plots from a single patient showing the gating strategy used to define the T cell populations assessed in Figure 4.
